# Establishment of Down’s syndrome periodontal ligament cells by transfection with *SV40T-Ag* and *hTERT*

**DOI:** 10.1007/s13577-021-00621-0

**Published:** 2021-09-29

**Authors:** Takeyoshi Asakawa, Atsushi Yamada, Masumi Kugino, Tomokazu Hasegawa, Kentaro Yoshimura, Kiyohito Sasa, Mitsuhiro Kinoshita, Masakazu Nitta, Karin Nagata, Tomomi Sugiyama, Ryutaro Kamijo, Takahiro Funatsu

**Affiliations:** 1 Departments of Pediatric Dentistry, School of Dentistry, Showa University, Tokyo, Japan; 2 Departments of Biochemistry, School of Dentistry, Showa University, Tokyo, Japan; 3 Department of Pediatric Dentistry, Institute of Health Bioscience, Tokushima University, Tokushima, Japan

**Keywords:** Periodontal ligament cells, Down’s syndrome, DSCR-1, *SV40T-Ag*, *hTERT*

## Abstract

Down’s syndrome is one of the most common human congenital genetic diseases and affected patients have increased risk of periodontal disease. To examine involvement of the disease with periodontal disease development, we established immortalized periodontal ligament cells obtained from a Down’s syndrome patient by use of *SV40T-Ag* and *hTERT* gene transfection. Expressions of *SV40T-Ag* and *hTERT* were observed in periodontal ligament cell-derived immortalized cells established from healthy (STPDL) and Down’s syndrome patient (STPDLDS) samples. Primary cultured periodontal ligament cells obtained from a healthy subject (pPDL) had a limited number of population doublings (< 40), while STPDL and STPDLDS cells continued to grow with more than 80 population doublings. Primary cultured periodontal ligament cells obtained from the patient showed a chromosome pattern characteristic of Down’s syndrome with trisomy 21, whereas STPDLDS samples showed a large number of abnormal chromosomes in those results. Gene expression analysis revealed that expression of *DSCR-1* in STPDLDS is greater than that in STPDL. These results suggest that the newly established STPDLDS cell line may be a useful tool for study of periodontal disease in Down’s syndrome patients.

**Supplementary Information:**

The online version contains supplementary material available at 10.1007/s13577-021-00621-0.

## Introduction

Down’s syndrome is one of the most frequently occurring congenital diseases among those caused by human chromosome abnormalities [1]. Affected patients have various congenital oral diseases, such as tooth absence, anatomical morphological tooth abnormalities, and delay in transformation from primary to permanent teeth [2]. Furthermore, they are susceptible to periodontal disease, which is the primary cause of early tooth loss and progress of the condition is difficult to control even with periodontology treatments [3–5]. Possible causes of Down’s syndrome patient susceptibility to periodontal disease include intellectual disability, making it difficult to perform daily mouth cleaning, and plaque or food remaining in the mouth, which is common in this population, though related factors remain unclear.

The periodontal ligament (PDL), established in mammals as part of the process of evolution, protects teeth even when the oral environment is harsh for a long period [6]. PDL tissue is composed primarily of cementum, osteoblasts, endothelial cells, epithelium of Malassez remnants, macrophages, and fibroblasts including osteoclasts and stem cells [7]. Recent studies have described methods for isolating PDL cells from periodontal tissues of primary and permanent teeth [8–10]. In PDL tissues found in periodontal pockets, inflammatory cytokines are produced by periodontal bacteria and physiological mechanical stress, factors known to cause periodontal disease and have great influence on the pathogenesis of periodontal disease [11]. We consider that a better understanding of the characteristics of PDL cells obtained from patients with Down’s syndrome will lead to new mechanisms of periodontal disease. In this study, immortalized periodontal ligament cells from patients with Down's syndrome were established and characterized.

## Materials and methods

### Periodontal ligament cell preparation and culture

Primary cultured periodontal ligament cells (pPDL) were established from the surface of a third molar extracted from a healthy 45-year-old male undergoing treatment. Healthy periodontal ligament cell-derived immortalized cells (STPDL) were obtained from the root surface of a first premolar extracted from an 11-year-old male undergoing orthodontic treatment. Primary (pPDLDS) and immortalized (STPDLDS) periodontal ligament cells were obtained from the root surface of a first premolar s extracted from an 11-year-old male with Down’s syndrome undergoing orthodontic treatment. All subjects visited the Dental Hospital of Showa University. The study protocol was approved by the Ethics committee of Showa University (No2013-007).

Extracted teeth were washed with α-modified minimum essential medium (α-MEM: Gibco BRL, Gaitherburg, MD, USA) containing 500 U/ml penicillin and 500 μg/ml streptomycin. We scraped the PDL from the root surface using a surgical blade and cut it into pieces for digestion with collagenase (2 mg/ml) at 37 °C for 30 min. Next, the pieces were washed with Dulbecco’s phosphate-buffered saline (PBS), placed into culture dishes, and maintained in α-MEM supplemented with 10% fetal bovine serum (FBS). Cells that grew from the cultures of PDL tissues obtained from the healthy male and boy, and Down’s syndrome patient were analyzed as pPDL, STPDL, STPDLDS, respectively.

#### Gene expression analysis based on microarray

Gene expression profiling was determined using an Affymetrix Human Clariom S Array (Life Technologies, Carlsbad, CA, USA). Data obtained in this study are publicly available in the Gene Expression Omnibus (GEO) database (accession number GSE143885). In GEO database, pPDL and pPDLDS are described as PDLGO and PDLDS, respectively. Gene ontology (GO) Slim analysis differentially expressed transcripts was also carried out.

#### Transfection of SV40T-Ag and hTERT genes

Primary PDL cells were transfected with a pBABE-puro-*SV40LT* plasmid (Addgene plasmid 13970; Addgene Inc. Cambrige, MA, USA) and pBABE-neo-*hTERT* plasmid containing a neomycin resistant gene (Addgene plasmid 1774; Addgene Inc. Cambrige, MA, USA) using a Lipofectamine™ LTX device (Invitrogen Life Technologies, Carlsbad, CA, USA), according to the manufacturer’s instruction. Cells were exposed to 10% FBS α-MEM containing 150 μg/ml G418 (Gibco BRL) and 0.5 μg/ml puromycin for 14 days. Surviving cells were trypsinized and allowed to grow in 100-mm culture dishes [12].

#### Short tandem repeat (STR) analysis

STR analysis was performed for the genetic signatures of 10 microsatellite markers using the Promega GenePrint 10 system.

#### Single-cell cloning

Single-cell clones were obtained using a limited dilution method. After transfection of the SV40T-Ag and hTERT genes, surviving cells in G418 and puromycin were seeded into a 96-well plate at 0.5 cells per well, then incubated at 37 °C in a humidified atmosphere of 95% air and 5% CO_2_. After the cells had grown for 14 days, they were treated with trypsin and subcultured in 60-mm culture dishes. The population doubling (PD) level was defined as the number of doublings required for a single cell to reach confluence in a 100-mm culture dish. Both the PD level and incubation day were considered to be zero when single-cell cloning was performed. The PD level of the PDL cell clones was estimated to be greater than 80and these cells were termed PDL for the present study.

#### Reverse transcription-polymerase chain reaction (RT-PCR)

One microgram of each RNA sample was reverse-transcribed to first-strand cDNA using a Prime Script RT reagent kit (Takara Bio, Inc., Shiga, Japan), according to the manufacturer’s protocol. Semi-quantitative PCR was performed with Taq polymerase (Promega, Madison, WI, USA) using specific PCR primers (Suppl. Fig. 1). Quantitative PCR was performed with SYBR Green Fast PCR Master Mix (Applied Biosystems, Waltham, MA, USA) using the following specific PCR primers: *DSCR-1*, 5’-TGCGACCCCAGTCATAAACTA-3’, and 5’-CCATTTCCTCTTCTTCCT-3’, *GAPDH*, 5’-GCACCGTCAAGGCTGAGAAC-3’, and 5’-TGGTGAAGACGCCAGTGGA-3’.

#### Immunohistochemical analysis of SV40T-Ag

For immunohistochemical analysis of the expression of *SV40T-Ag*, cells were fixed with 10% formaldehyde, then washed with PBS and incubated with mouse anti- *SV40T-Ag* (Ab-2, Calbiochem, CA, USA). The reaction products were visualized using an AEC substrate kit (Nichirei, Tokyo, Japan).

#### hTERT expression analysis

*hTERT* expression analysis was conducted with an enzyme-linked immunosorbent assay (ELISA) using an Ab-Match Assembly Human TERT Kit (MBL, Nagoya, Aichi, Japan), according to the manufacturer’s protocol [13].

#### Karyotyping analysis

All karyotyping analyses were performed by Chromocenter Inc. (Yanago, Shiga, Japan). Preparation of metaphase chromosomes of cells was performed according to standard methods [14].

## Results and discussion

**Fig. 1:**
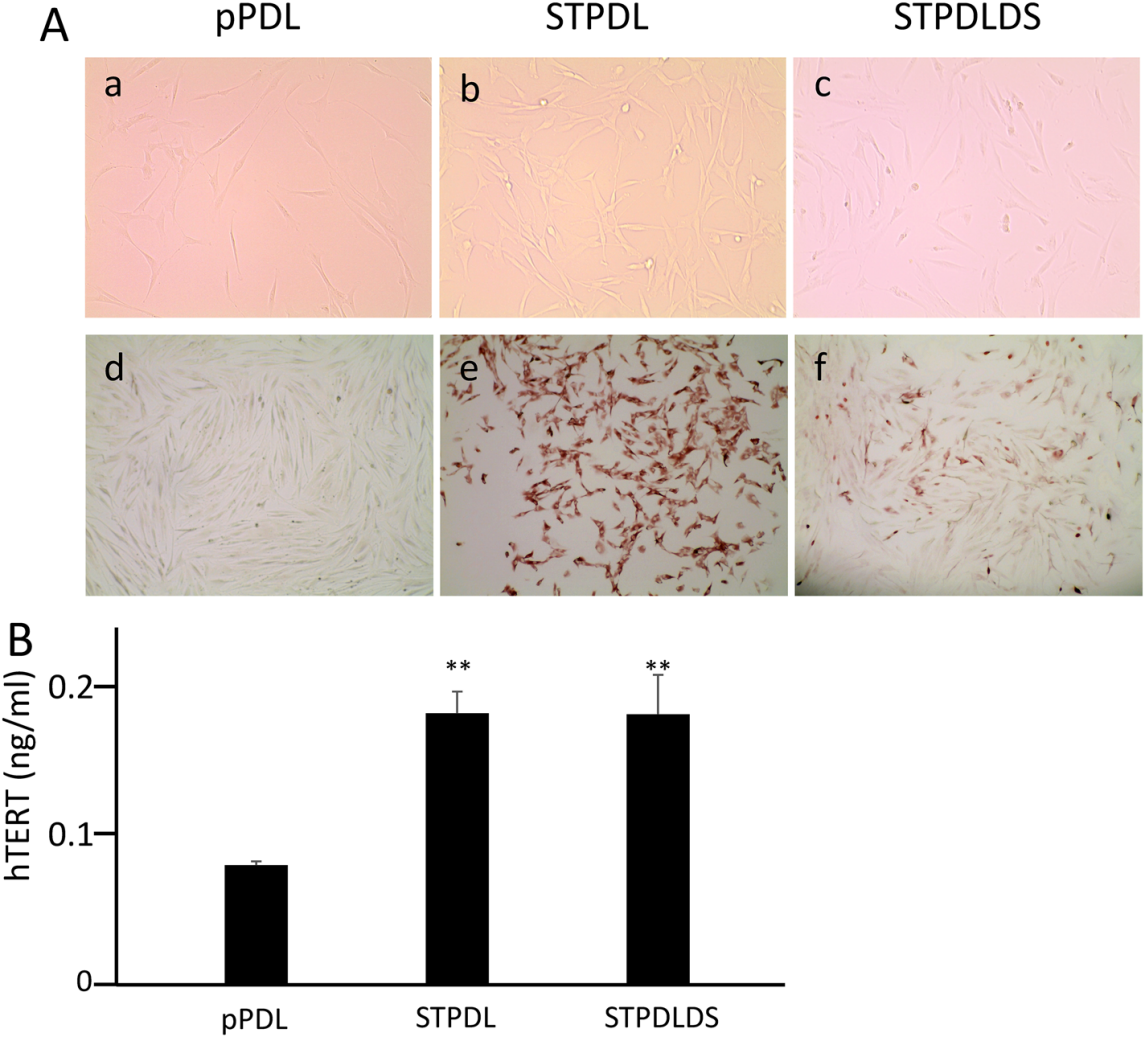
**A** Representative images of pPDL (**a**), STPDL (**b**) and STPDLDS (**c**) obtained with an optical microscope. Immunohistochemical staining for *SV40T-Ag* was positive in STPDL (**e**) and STPDLDS (**f**), but not pPDL (**d**). **B** Levels of *hTERT* protein expression determined by ELISA. Each experiment was performed at least three times and values are shown as the mean ± SD. **P < 0.01, Students, *t* test

**Fig. 2:**
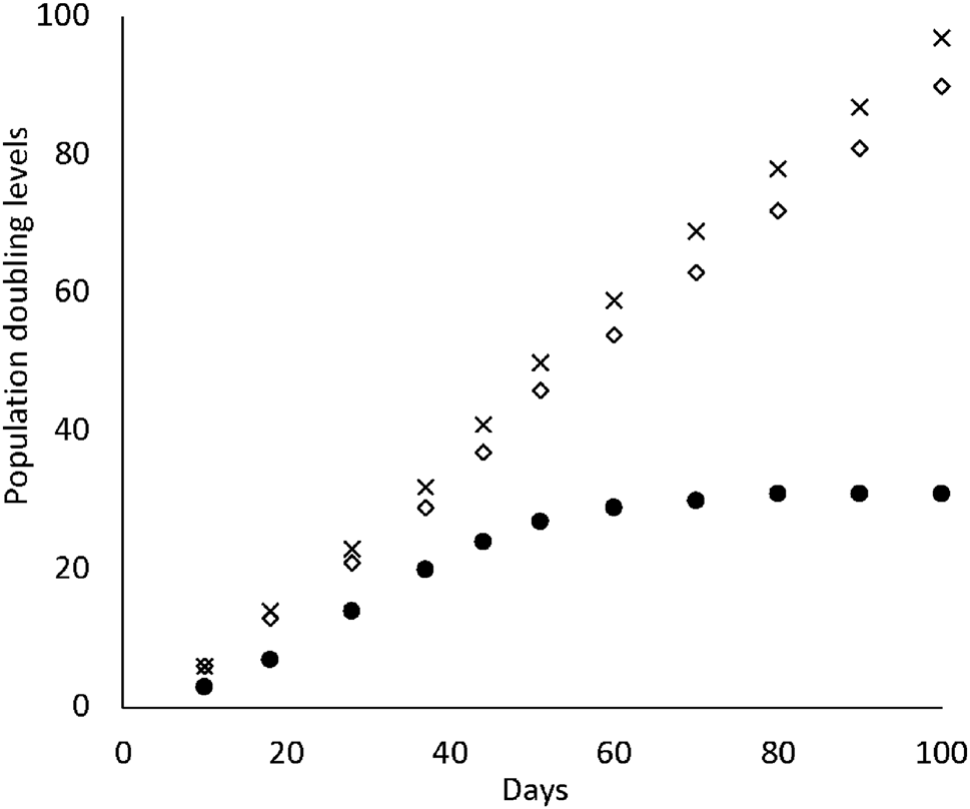
Population doubling (PD) of (●) pPDL, (◇) STPDL, and (×) STPDLDS. The *x* axis indicates incubation days and the *y* axis PD number

For the present study, we initially obtained periodontal ligament cells from healthy subjects as well as a patient with Down’s syndrome. Examination of primary cultured periodontal ligament cells from the patient using karyotyping analysis revealed a chromosome pattern characteristic of Down’s syndrome with 3 occurrences of chromosome 21 (Suppl. Fig. 2A). In addition, microarray analysis of the gene expression patterns of pPDL and pPDLDS revealed a characteristic gene expression pattern in those from the patient (Suppl. Table 1, 2). GO slim analysis was carried out about the Biological Process, the Cellular Component, and the Molecular Function, and some differences were noted between up- and down-regulated genes in pPDLDS (Suppl. Fig. 3). Thus, we attempted to establish immortalized cells by introduction of the *SV40T-Ag* and *hTERT* genes. There were no differences in cell morphology observed among the pPDL, STPDL (*SV40T-Ag* and *hTERT* transfected periodontal ligament cells), and STPDLDS (*SV40T-Ag* and *hTERT* transfected periodontal ligament cells from Down’s syndrome patient) samples (Fig. 1Aa–c). Short tandem repeat (STR) analysis demonstrated that the STPDLDS sample matched the fibroblasts in the volunteer sample (Evaluation Value (EV) = 0.909) (Suppl. Table 3). When expression of SV40T-Ag was examined by cellular immunostaining, that was observed in the STPDL and STPDLDS, but not the pPDL cells (Fig. 1Ad–f), while the expression of *hTERT* protein, examined by ELISA, was significantly increased in STPDL and STPDLDS as compared with pPDL. To confirm that the STPDL and STPDLDS cells had become immortalized, the cell doubling (PD) number was examined. In pPDL, PD occurred less than 40 times, while that was greater than 80 times in STPDL and STPDLDS (Fig. 2). Based on these results, we considered that immortalized cells could be established from periodontal ligament cells of healthy subjects as well as patients with Down’s syndrome. Karyotyping analysis of STPDLDS revealed a large number of abnormal chromosomes [15, 16] (Suppl. Fig. 2B).

**Fig. 3:**
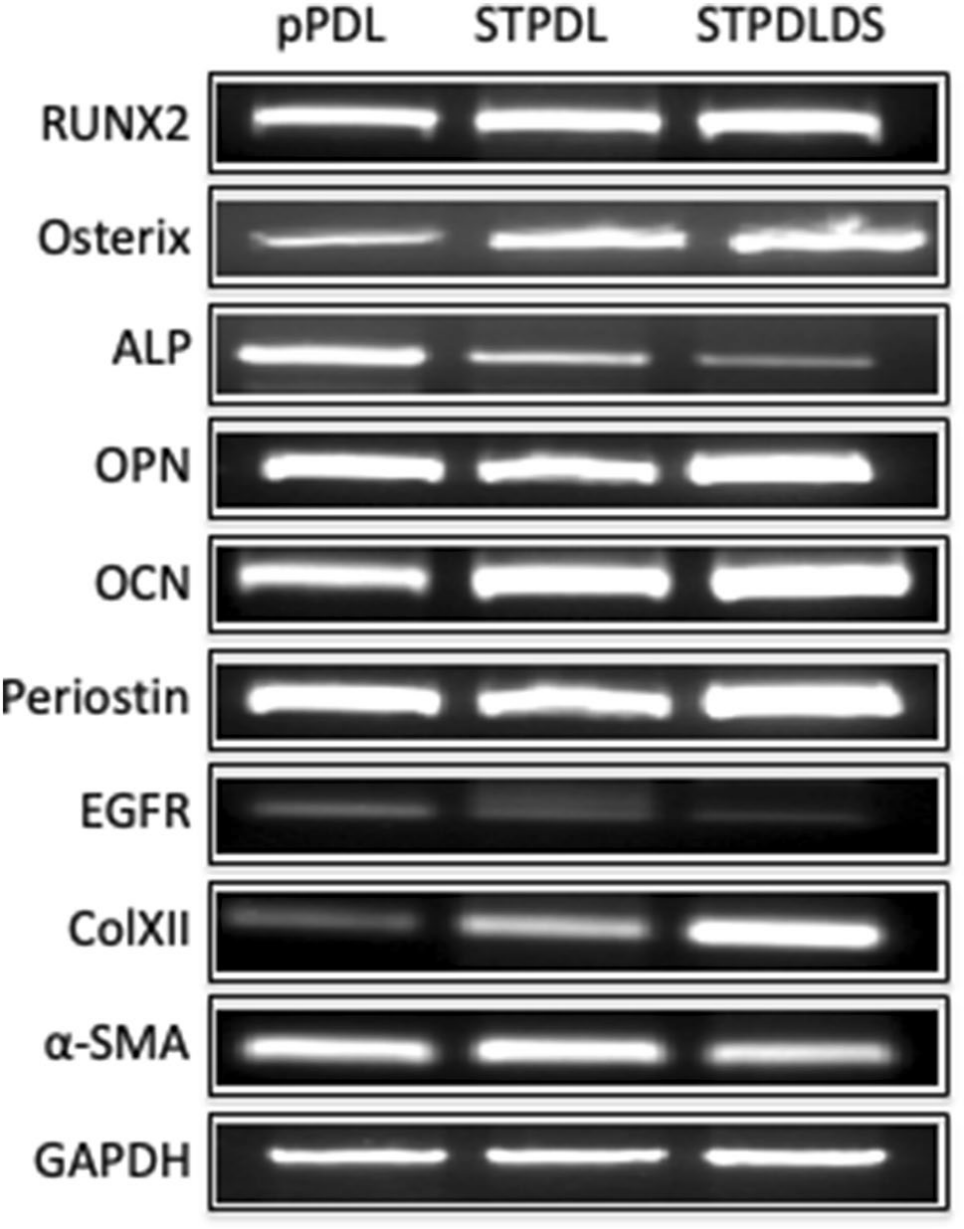
Expressions of RUNX2, Osterix, ALP, OPN, OCN, Periostin, EGFR, ColXII, α-SMA, and GAPDH in pPDL, STPDL and STPDLDS, as shown by semi-quantitative reverse transcription PCR analysis results

**Fig. 4:**
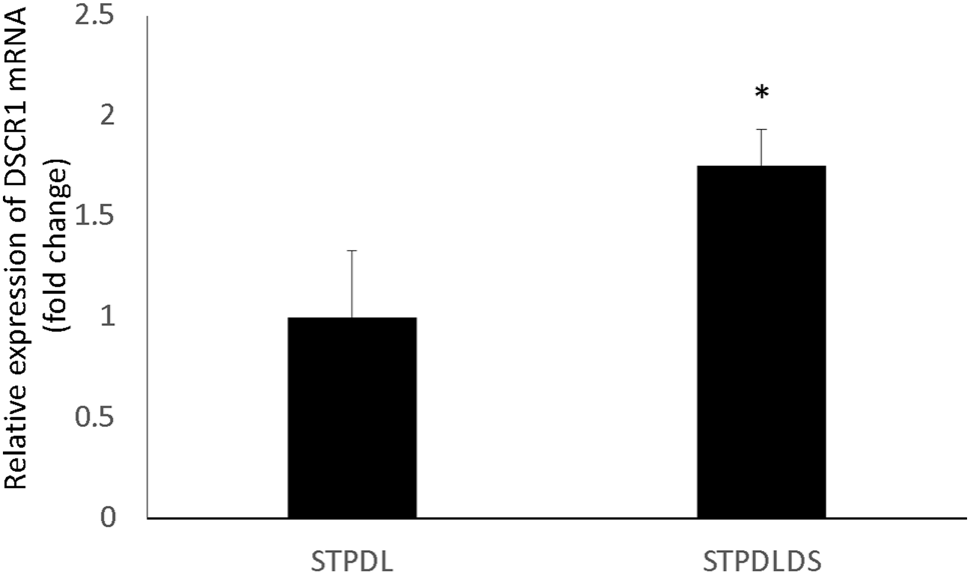
Expression of DSCR1 in STPDLDS was higher than that in STPDL. Total cellular RNA was extracted and mRNA levels of DSCR1 and GAPDH were examined using quantitative real time PCR analysis. Results are shown as the mean ± SD of 3 samples. *P < 0.05 student’s *t* test

Next, the gene expression patterns of STPDL and STPDLDS were examined, and expressions of characteristic genes in those cells, such as *RUNX2, Osterix, ALP, OPN, OCN, Periostin, EGFR, ColXII, and α-SMA,* were noted (Fig. 3). Furthermore, expression of *DSCR-1*, which is found on human chromosome 21 and encodes a protein that suppresses vascular endothelial growth factor mediated angiogenic signaling [17], was examined in STPDL and STPDLDS, and found to be greater in the latter, consistent with our cDNA microarray analysis results (Fig. 4). Together, these findings suggest that the newly established STPDLDS may be a useful tool for investigation of periodontal disease in patients with Down’s syndrome.

*DSCR-1* expression is known to be enhanced in cells derived from Down’s syndrome patients [18]. Furthermore, various biological activities are exhibited when intracellular transduction through calcineurin, which interacts with *DSCR-1*, is inhibited [19]. Also, *DSCR-1* suppresses vascular endothelial growth factor (*VEGF*)-mediated angiogenic signaling via the calcineurin pathway [20]. We speculate that suppression of angiogenesis by *DSCR-1* makes it difficult to treat periodontal disease. In the future, it is anticipated that the role of *DSCR-1* in PDL cells of patients with Down’s syndrome can be elucidated by use of STPDLDS, with the findings useful to clarify the cause of acute progressive periodontal disease often observed in those patients.

Angiogenesis is a process that supplies nutrients and oxygen to tissues, and is considered to be deeply involved in wound healing in periodontium tissues as well as metastasis in malignant tumors [21]. However, several details regarding the associated mechanisms remain unclear. In individuals with Down’s syndrome, suppression of angiogenic ability has been observed by enhancement of the activity of DSCR-1 and inhibiting intracellular signal transduction via calcineurin. As a result, the risk of death from a solid tumor is only approximately 10% as compared to that in individuals without Down’s syndrome due to suppression of angiogenesis [19]. We consider that angiogenesis suppression caused by activation of DSCR-1 makes it wound healing difficult in Down's syndrome patients and expect the STPDLDS cell line to be a useful tool for research of periodontal tissue wound healing.

To summarize, the present findings indicate that newly established STPDLDS can be a useful tool for the study of periodontal disease in Down’s syndrome.

## Supplementary Information

Below is the link to the electronic supplementary material.

Suppl. Fig. 1 Primer sequences for semi-quantitative reverse transcription PCR. (TIFF 19 kb)

Suppl. Fig. 2 Karyotyping analysis of (A) primary cultured periodontal ligament cells from Down’s syndrome patients and (B) STPDLDS. (TIFF 3571 kb)

Suppl. Fig. 3 Gene ontology (GO) slim analysis using (A) the Biological Process, (B) the Cellular Component, and (C) the Molecular Function in up-regulated (a) and down-regulated (b) genes in pPDLDS (TIFF 2621 kb)

Supplementary file4 (PDF 59 kb)

Supplementary file5 (PDF 66 kb)

Supplementary file6 (TIFF 6 kb)
