# Supplementary figures and images for: Establishment of Down’s syndrome periodontal ligament cells by transfection with SV40T-Ag and hTERT
Source: Hum Cell. 2021 Sep 29;35(1):379–83. doi: 10.1007/s13577-021-00621-0 (PMC8732922; doi:10.1007/s13577-021-00621-0)

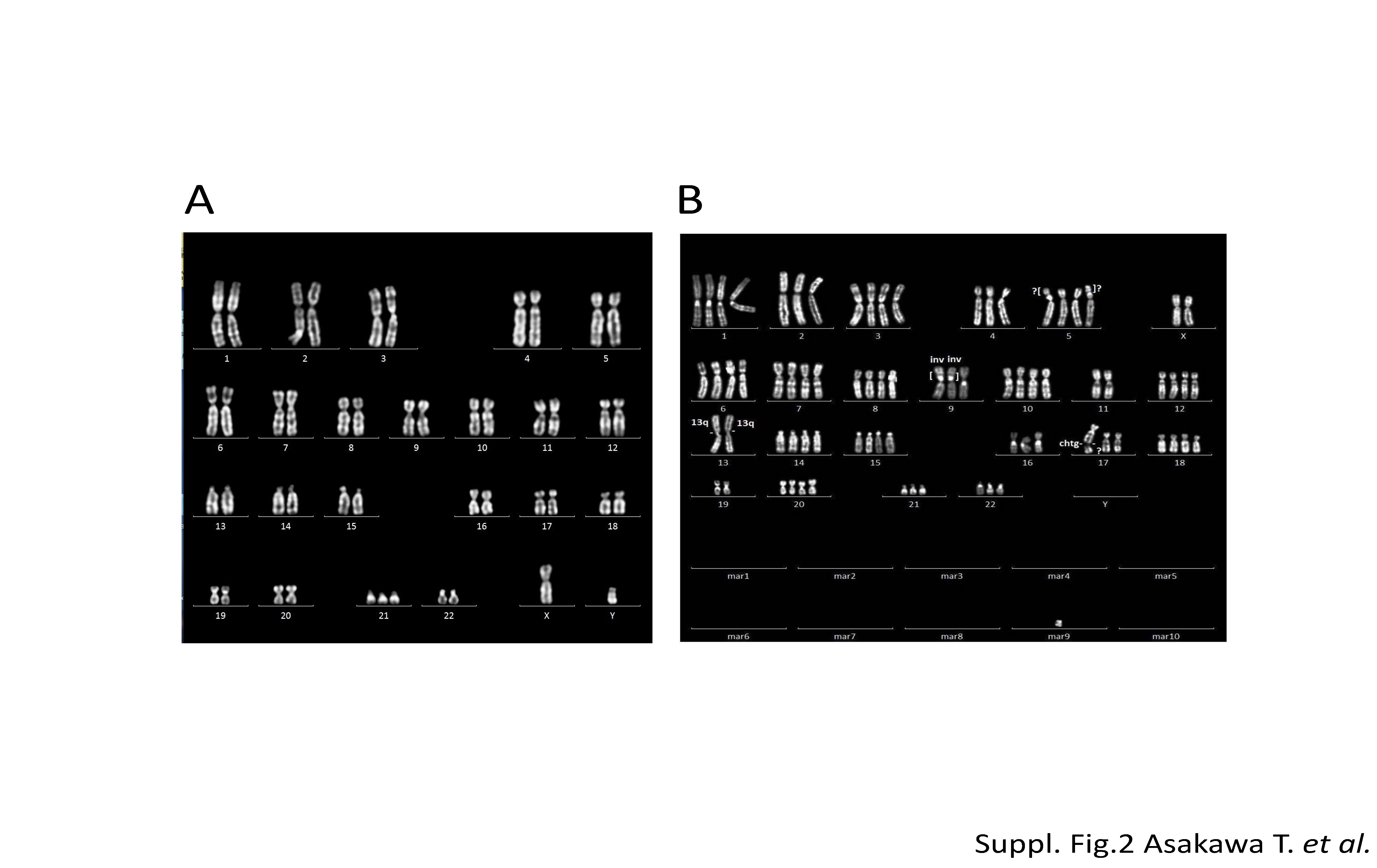

Supplement: Supplementary file 2 — Suppl. Fig. 2 Karyotyping analysis of (A) primary cultured periodontal ligament cells from Down’s syndrome patients and (B) STPDLDS. (TIFF 3571 kb) [file 13577_2021_621_MOESM2_ESM.tiff]

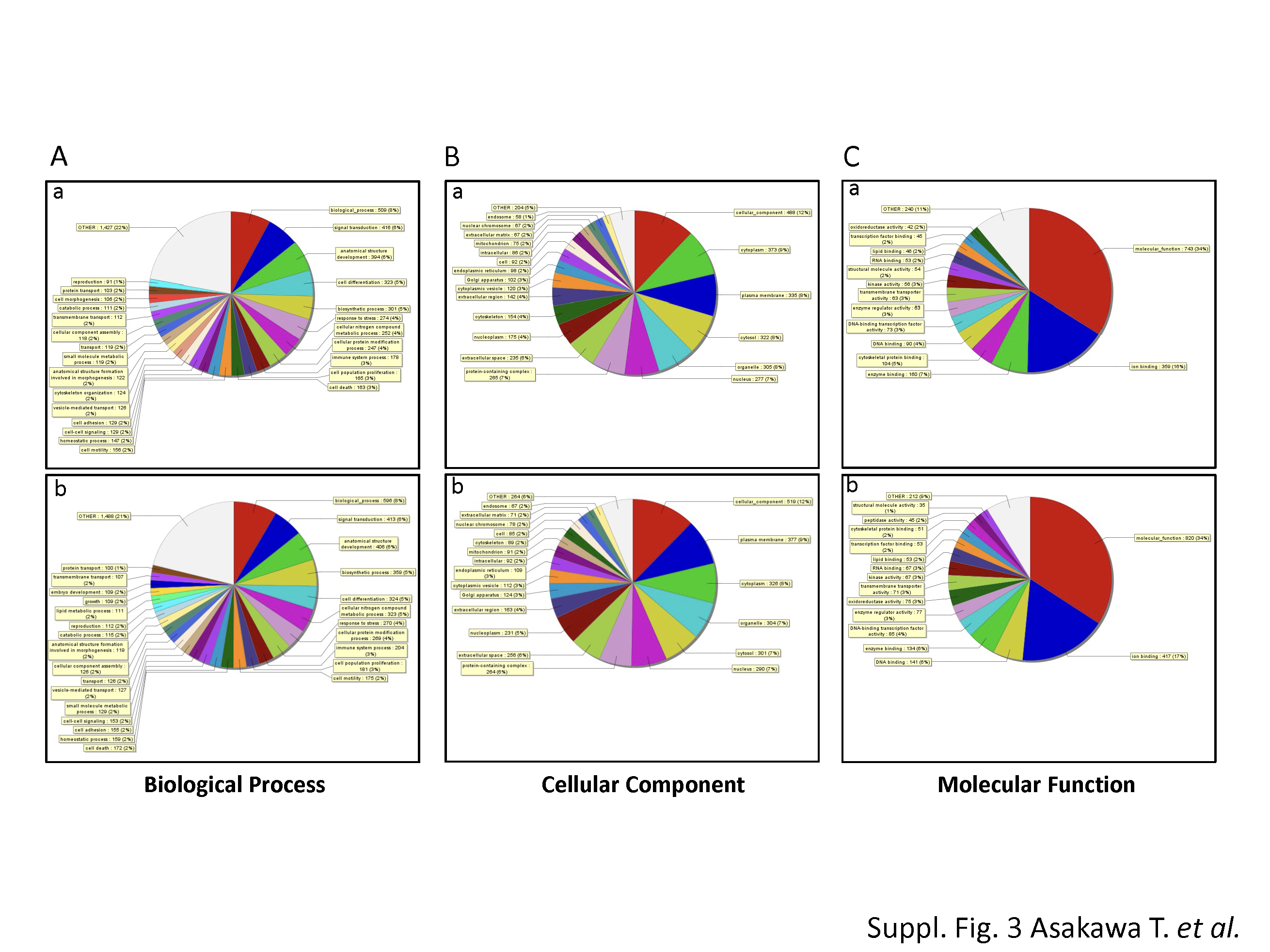

Supplement: Supplementary file 3 — Suppl. Fig. 3 Gene ontology (GO) slim analysis using (A) the Biological Process, (B) the Cellular Component, and (C) the Molecular Function in up-regulated (a) and down-regulated (b) genes in pPDLDS (TIFF 2621 kb) [file 13577_2021_621_MOESM3_ESM.tiff]
