# Supplementary material for: Establishment of Down’s syndrome periodontal ligament cells by transfection with SV40T-Ag and hTERT
Source: Hum Cell. 2021 Sep 29;35(1):379–83. doi: 10.1007/s13577-021-00621-0 (PMC8732922; doi:10.1007/s13577-021-00621-0)
Supplement: Supplementary file 4 — Supplementary file4 (PDF 59 kb) [file 13577_2021_621_MOESM4_ESM.pdf]

Suppl. Table 1

Highly up-regulated gene profiles in pPDLDS compared with those in pPDL. FC; fold change

log_2FC	Gene symbol	Gene name
10.713724	PLPPR4	phospholipid phosphatase related 4
9.7110827	MYOCD	myocardin
9.5428366	SCUBE3	signal peptide, CUB domain, EGF-like 3
9.4982485	TINAGL1	tubulointerstitial nephritis antigen-like 1
9.4122466	RGS4	regulator of G-protein signaling 4
9.2558369	NEFM	neurofilament, medium polypeptide
9.0061986	COL4A1	collagen, type IV, alpha 1
7.7911409	ANKRD1	ankyrin repeat domain 1 (cardiac muscle)
7.3068294	NDUFA4L2	NADH dehydrogenase (ubiquinone) 1 alpha subcomplex, 4-like 2
7.2637465	KCNMB1	potassium channel subfamily M regulatory beta subunit 1
6.9665182	OLFM2	olfactomedin 2
6.7002244	TFPI2	tissue factor pathway inhibitor 2
6.6991884	AQP1	aquaporin 1 (Colton blood group)
6.5601158	NREP	neuronal regeneration related protein
6.3211603	ACTG2	actin, gamma 2, smooth muscle, enteric
6.2188302	EDN1	endothelin 1
6.1334728	HEY2	hes-related family bHLH transcription factor with YRPW motif 2
6.0786464	TM4SF1	transmembrane 4 L six family member 1
5.9206319	MYH11	myosin, heavy chain 11, smooth muscle
5.8769055	CORIN	corin, serine peptidase
5.8655127	ASB5	ankyrin repeat and SOCS box containing 5
5.8497921	BEX1	brain expressed X-linked 1
5.5652734	DSP	desmoplakin
5.5334173	SUSD2	sushi domain containing 2
5.5128955	SLC2A1	solute carrier family 2 (facilitated glucose transporter), member 1
5.4852246	TRPC6	transient receptor potential cation channel, subfamily C, member 6
5.3903184	ERAP2	endoplasmic reticulum aminopeptidase 2
5.3860451	CABP1	calcium binding protein 1
5.3727241	PPP1R14A	protein phosphatase 1, regulatory (inhibitor) subunit 14A
5.2167184	EPHA5	EPH receptor A5
5.1849407	PDGFA	platelet-derived growth factor alpha polypeptide
5.1724628	ACAN	aggrecan
5.1375902	PDE1C	phosphodiesterase 1C, calmodulin-dependent 70kDa
5.1171116	JAG1	jagged 1
4.9746916	NOTCH3	notch 3
4.9602396	NTN4	netrin 4
4.9566313	FGD4	FYVE, RhoGEF and PH domain containing 4
4.9481512	PLPP4	phospholipid phosphatase 4
4.8428018	POSTN	periostin, osteoblast specific factor
4.819836	MCAM; MIR6756	melanoma cell adhesion molecule; microRNA 6756
4.7844218	LRRC32	leucine rich repeat containing 32
4.7629447	AK4	adenylate kinase 4
4.7446225	A2M	alpha-2-macroglobulin
4.6540375	DAAM2	dishevelled associated activator of morphogenesis 2
4.6051907	SHC4	SHC (Src homology 2 domain containing) family, member 4
4.5797728	PDK1	pyruvate dehydrogenase kinase, isozyme 1
4.5179195	COL4A2	collagen, type IV, alpha 2
4.4618015	EBF2	early B-cell factor 2
4.4565746	TGFB2; TGFB2-OT1	transforming growth factor beta 2; TGFB2 overlapping transcript 1
4.4009249	TRPC4	transient receptor potential cation channel, subfamily C, member 4
4.3966313	CTPS1	CTP synthase 1
4.3574807	CCL28	chemokine (C-C motif) ligand 28
4.3357696	INHBA	inhibin beta A
4.3080842	LBH	limb bud and heart development
4.284939	PAG1	phosphoprotein membrane anchor with glycosphingolipid microdomains 1
4.2836134	KCNE4	potassium channel, voltage gated subfamily E regulatory beta subunit 4
4.2643355	BHLHE40	basic helix-loop-helix family, member e40
4.2165682	PLAT	plasminogen activator, tissue
4.2142601	SPOCD1	SPOC domain containing 1
4.2026703	BGN	biglycan
4.1836055	GPR183	G protein-coupled receptor 183
4.167016	HLA-DRB1	major histocompatibility complex, class II, DR beta 1
4.1064781	ITGA3	integrin alpha 3
4.0758772	ID3	inhibitor of DNA binding 3, dominant negative helix-loop-helix protein
4.0596882	KCTD20	potassium channel tetramerization domain containing 20
4.0518968	GYS1	glycogen synthase 1 (muscle)
4.0059532	ANK3	ankyrin 3, node of Ranvier (ankyrin G)
3.9848192	PDE3A	phosphodiesterase 3A, cGMP-inhibited
3.9654231	STC2	stanniocalcin 2
3.9569861	STK38L	serine/threonine kinase 38 like
3.947504	TNC	tenascin C
3.9169387	NRXN3	neurexin 3
3.8952031	EFHD1	EF-hand domain family member D1
3.8520067	IER3	immediate early response 3
3.8038143	ITGA7	integrin alpha 7
3.7983457	TES	testin LIM domain protein
3.7823752	OXTR	oxytocin receptor
3.655654	RELN	reelin
3.6497866	HLA-DRB5	major histocompatibility complex, class II, DR beta 5
3.6317582	RNF182	ring finger protein 182
3.6314566	IFI44L	interferon-induced protein 44-like
3.6291197	ZNF423	zinc finger protein 423
3.6233637	GNA14	guanine nucleotide binding protein (G protein), alpha 14
3.6084629	NR4A3	nuclear receptor subfamily 4, group A, member 3
3.5864831	PGK1	phosphoglycerate kinase 1
3.5721261	RARB	retinoic acid receptor, beta
3.5695895	TGFBI	transforming growth factor, beta-induced, 68kDa
3.567276	IFI27	interferon, alpha-inducible protein 27
3.5604191	OLFML2B	olfactomedin like 2B
3.5465929	NETO2	neuropilin (NRP) and tolloid (TLL)-like 2
3.5210989	CSRP2	cysteine and glycine-rich protein 2
3.5112506	C10orf10	chromosome 10 open reading frame 10
3.4993462	CCDC81	coiled-coil domain containing 81
3.4733699	NR2F2	nuclear receptor subfamily 2, group F, member 2
3.4616601	PLXDC1	plexin domain containing 1
3.4595644	WFDC1	WAP four-disulfide core domain 1
3.4498766	COL18A1	collagen, type XVIII, alpha 1
3.4495401	CNN1	calponin 1, basic, smooth muscle
3.4346948	ENTPD1	ectonucleoside triphosphate diphosphohydrolase 1
3.4285622	HSPB7	heat shock 27kDa protein family, member 7 (cardiovascular)
3.412727	CSPG4	chondroitin sulfate proteoglycan 4
3.4063643	KCTD16	potassium channel tetramerization domain containing 16
3.3711797	IFI6	interferon, alpha-inducible protein 6
3.3645646	LGMN	legumain
3.3620508	TNFAIP3	tumor necrosis factor, alpha-induced protein 3
3.360587	CPNE4	copine IV
3.3491058	STC1	stanniocalcin 1
3.3457204	ABLIM1	actin binding LIM protein 1
3.3329282	PCDH7	protocadherin 7
3.3231983	LGR5	leucine-rich repeat containing G protein-coupled receptor 5
3.3117741	HHIP	hedgehog interacting protein
3.3031144	NES	nestin
3.2937229	CD4	CD4 molecule
3.2825239	ELN	elastin
3.2422463	ERRFI1	ERBB receptor feedback inhibitor 1
3.2315822	C3orf70	chromosome 3 open reading frame 70
3.2074216	ITIH3	inter-alpha-trypsin inhibitor heavy chain 3
3.2069077	PDK3	pyruvate dehydrogenase kinase, isozyme 3
3.1960837	MGAM	maltase-glucoamylase
3.1920037	PFKP	phosphofructokinase, platelet
3.1598395	SRGN	serglycin
3.1390746	ARHGAP24	Rho GTPase activating protein 24
3.1280503	RASGRP1	RAS guanyl releasing protein 1 (calcium and DAG-regulated)
3.1218947	IGFBP3	insulin like growth factor binding protein 3
3.1218225	PFKL	phosphofructokinase, liver
3.1206628	C2CD2	C2 calcium-dependent domain containing 2
3.1197865	SIK1	salt-inducible kinase 1
3.1127885	LAMA1	laminin, alpha 1
3.0995118	SRGN	Memczak2013 ANTISENSE, coding, INTERNAL, UTR3 best transcript NM_002727
3.0935582	PALMD	palmdelphin
3.0934013	SLC1A1	solute carrier family 1 member 1
3.084919	ITGB1BP2	integrin beta 1 binding protein (melusin) 2
3.0631229	MEDAG	mesenteric estrogen-dependent adipogenesis
3.0582486	HSPA2	heat shock 70kDa protein 2
3.039671	PFKFB4; MIR6823	6-phosphofructo-2-kinase/fructose-2,6-biphosphatase 4; microRNA 6823
3.0388384	RHOJ	ras homolog family member J
3.0216164	KCNK6	potassium channel, two pore domain subfamily K, member 6
3.0117804	COL5A1	collagen, type V, alpha 1
3.0103281	SDC2	syndecan 2
3.006914	RCAN1	regulator of calcineurin 1
3.0053158	PTPRJ	protein tyrosine phosphatase, receptor type, J
3.0049839	MICAL2	microtubule associated monooxygenase, calponin and LIM domain containing 2
2.9955254	NR4A2	nuclear receptor subfamily 4, group A, member 2
2.9885562	FOXC2	forkhead box C2
2.9880903	PARP12	poly(ADP-ribose) polymerase family member 12
2.9862592	MIR6809; TNS1	microRNA 6809; tensin 1
2.9774169	JPH2	junctophilin 2
2.9752028	MLLT11	myeloid/lymphoid or mixed-lineage leukemia; translocated to, 11
2.9733905	TDRD1	tudor domain containing 1
2.9062593	ATP1B1	ATPase, Na+/K+ transporting, beta 1 polypeptide
2.8971347	MYO1E	myosin IE
2.8909134	SPHK1	sphingosine kinase 1
2.8841363	FRMPD4	FERM and PDZ domain containing 4
2.8565848	SH3RF1	SH3 domain containing ring finger 1
2.8373091	PRR5L	proline rich 5 like
2.8362063	CRHBP	corticotropin releasing hormone binding protein
2.8328131	TGFB3	transforming growth factor beta 3
2.8150366	UBASH3B	ubiquitin associated and SH3 domain containing B
2.814232	SEMA7A	semaphorin 7A, GPI membrane anchor (John Milton Hagen blood group)
