# Supplementary material for: Establishment of Down’s syndrome periodontal ligament cells by transfection with SV40T-Ag and hTERT
Source: Hum Cell. 2021 Sep 29;35(1):379–83. doi: 10.1007/s13577-021-00621-0 (PMC8732922; doi:10.1007/s13577-021-00621-0)
Supplement: Supplementary file 5 — Supplementary file5 (PDF 66 kb) [file 13577_2021_621_MOESM5_ESM.pdf]

Suppl. Table 2

Highly down-regulated gene profiles in pPDLDS compared with those in pPDL. FC; fold change

$\log_2$FC	Gene symbol	Gene name
−10.10928551	PENK	proenkephalin
−9.585372579	CPXM2	carboxypeptidase X (M14 family), member 2
−7.526934346	LEPR; LEPROT	leptin receptor; leptin receptor overlapping transcript
−7.249424506	PTGIS	prostaglandin I2 (prostacyclin) synthase
−6.915309737	SLC14A1	solute carrier family 14 (urea transporter), member 1 (Kidd blood group)
−6.653333811	OMD	osteomodulin
−6.608220437	MME	membrane metallo-endopeptidase
−6.427976109	ITGB8	integrin beta 8
−6.246451645	COLEC12	collectin sub-family member 12
−6.190915023	GPC6	glypican 6
−6.179787792	PCDH10	protocadherin 10
−6.093289219	FLRT2	fibronectin leucine rich transmembrane protein 2
−6.07769019	BMP4	bone morphogenetic protein 4
−5.750445226	NOVA1	neuro-oncological ventral antigen 1
−5.746943321	TM4SF20	transmembrane 4 L six family member 20
−5.650541446	MFAP4	microfibrillar associated protein 4
−5.604431151	SLC9A9	solute carrier family 9, subfamily A (NHE9, cation proton antiporter 9), member 9
−5.604125427	MKX	mohawk homeobox
−5.476787404	PRSS12	protease, serine, 12 (neurotrypsin, motopsin)
−5.461292272	PPAP2B	Transcript Identified by AceView, Entrez Gene ID(s) 8613
−5.45748978	GREM2	gremlin 2, DAN family BMP antagonist
−5.278614078	F2RL2	coagulation factor II (thrombin) receptor-like 2
−5.264536925	ISLR	immunoglobulin superfamily containing leucine-rich repeat
−5.263862938	PLPP3	phospholipid phosphatase 3
−5.245937662	ENPP2	ectonucleotide pyrophosphatase/phosphodiesterase 2
−5.22851695	HS3ST3B1	heparan sulfate (glucosamine) 3-O-sulfotransferase 3B1
−5.184165646	CPE	carboxypeptidase E
−5.169725253	LSAMP	limbic system-associated membrane protein
−5.043723129	DOK6	docking protein 6
−5.030363007	SLC7A8	solute carrier family 7 (amino acid transporter light chain, L system), member 8
−5.023671894	MN1	meningioma (disrupted in balanced translocation) 1
−4.938647942	RDH10	retinol dehydrogenase 10 (all-trans)
−4.893524305	TMTC1	transmembrane and tetratricopeptide repeat containing 1
−4.849637495	GMNC	geminin coiled-coil domain containing
−4.823145354	PDGFRA	platelet-derived growth factor receptor, alpha polypeptide
−4.747126143	ALDH1A1	aldehyde dehydrogenase 1 family, member A1
−4.725627606	EYA4	EYA transcriptional coactivator and phosphatase 4
−4.706287522	TNFRSF11B	tumor necrosis factor receptor superfamily, member 11b
−4.69578589	ABCA8	ATP binding cassette subfamily A member 8
−4.674133556	TNFRSF19	tumor necrosis factor receptor superfamily, member 19
−4.602360015	SVEP1	sushi, von Willebrand factor type A, EGF and pentraxin domain containing 1
−4.599761474	RASGRF2	Ras protein-specific guanine nucleotide-releasing factor 2
−4.591959188	COL12A1	collagen, type XII, alpha 1
−4.545818449	GALNT14	polypeptide N-acetylgalactosaminyltransferase 14
−4.520131934	DHCR24	24-dehydrocholesterol reductase
−4.458366569	TOX	thymocyte selection-associated high mobility group box
−4.441824807	CACNA2D3	calcium channel, voltage-dependent, alpha 2/delta subunit 3
−4.378991379	SLC2A12	solute carrier family 2 (facilitated glucose transporter), member 12
−4.359462815	MGAT5	mannosyl (alpha-1,6-)-glycoprotein beta-1,6-N-acetyl-glucosaminyltransferase
−4.35085489	APBB1IP	amyloid beta (A4) precursor protein-binding, family B, member 1 interacting protein
−4.310800527	STEAP2	STEAP family member 2, metalloreductase
−4.283943108	FBLN1	fibulin 1
−4.275406908	PDPN	podoplanin
−4.261253498	IGF2	insulin-like growth factor 2
−4.214439446	FGF7	fibroblast growth factor 7
−4.208818278	AHNAK2	AHNAK nucleoprotein 2
−4.170955121	MXRA5	matrix-remodelling associated 5
−4.127232176	AMOT; MIR4329	angiomotin; microRNA 4329
−4.02153379	MID1	midline 1
−4.012037305	HAPLN1	hyaluronan and proteoglycan link protein 1
−3.993831602	CAPG	capping protein (actin filament), gelsolin-like
−3.973912255	XG; XGY2	Xg blood group; Xg pseudogene, Y-linked 2
−3.956670163	NTNG1	netrin G1
−3.946809857	MOXD1	monooxygenase, DBH-like 1
−3.909325604	MGST1	microsomal glutathione S-transferase 1
−3.899913663	RUNX2	runt-related transcription factor 2
−3.879392667	ADAMTSL1	ADAMTS like 1
−3.86849931	SPON1	spondin 1, extracellular matrix protein
−3.855955137	EPHX1	epoxide hydrolase 1, microsomal (xenobiotic)
−3.826568594	GULP1	GULP, engulfment adaptor PTB domain containing 1
−3.812143128	EFNA5	ephrin-A5
−3.78173645	SERPINF1	serpin peptidase inhibitor, clade F member 1
−3.742844516	SEPP1	selenoprotein P, plasma, 1
−3.720046916	RHOBTB3	Rho-related BTB domain containing 3
−3.70229125	FADS2	fatty acid desaturase 2
−3.690784637	EYA1	EYA transcriptional coactivator and phosphatase 1
−3.623077429	C1orf21	chromosome 1 open reading frame 21
−3.602317516	ADAMTS3	ADAM metallopeptidase with thrombospondin type 1 motif 3
−3.579988904	MOCOS	molybdenum cofactor sulfurase
−3.569185265	HS3ST3A1	heparan sulfate (glucosamine) 3-O-sulfotransferase 3A1
−3.558330699	SH2D4A	SH2 domain containing 4A
−3.550039405	C1S	complement component 1, s subcomponent
−3.531088127	MASP1	mannan-binding lectin serine peptidase 1
−3.514263346	MBOAT1	membrane bound O-acyltransferase domain containing 1
−3.508272175	SULF2	sulfatase 2
−3.49136227	OGN	osteoglycin
−3.478931779	PDGFD	platelet derived growth factor D
−3.468848829	ST8SIA1	ST8 alpha-N-acetyl-neuraminide alpha-2,8-sialyltransferase 1
−3.440979621	PTGFR	prostaglandin F receptor (FP)
−3.432463587	RP11-307P5.1; SAMD5	novel transcript; Transcript Identified by AceView, Entrez Gene ID(s) 389432
−3.430314851	SLC25A27	solute carrier family 25, member 27
−3.430212036	VCAM1	vascular cell adhesion molecule 1
−3.406185703	ANGPTL2	angiopoietin like 2
−3.404776606	CNTN3	contactin 3 (plasmacytoma associated)
−3.40141115	ANKRD29	ankyrin repeat domain 29
−3.389928889	FBN2	fibrillin 2
−3.387025753	PLXDC2	plexin domain containing 2
−3.368853643	C3	complement component 3
−3.360803494	BNC2	basonuclin 2
−3.354972806	CLU; MIR6843	clusterin; microRNA 6843
−3.335321438	AKR1C1	aldo-keto reductase family 1, member C1
−3.324081473	IGF2; INS-IGF2	insulin-like growth factor 2; INS-IGF2 readthrough
−3.314794424	CDK14	cyclin-dependent kinase 14
−3.298190712	TNFSF15	tumor necrosis factor (ligand) superfamily, member 15
−3.29088596	PLSCR4	phospholipid scramblase 4
−3.289840698	TRIM2	tripartite motif containing 2
−3.264903948	CD55	CD55 molecule, decay accelerating factor for complement (Cromer blood group)
−3.260154509	DHRS3; MIR6730	dehydrogenase/reductase (SDR family) member 3; microRNA 6730
−3.257108744	ZEB1	zinc finger E-box binding homeobox 1
−3.247074262	EPB41L3	erythrocyte membrane protein band 4.1-like 3
−3.23513429	CTSK	cathepsin K
−3.217921863	GABBR2	gamma-aminobutyric acid (GABA) B receptor, 2
−3.210559764	SFRP1	secreted frizzled-related protein 1
−3.203674176	OR4F15	olfactory receptor, family 4, subfamily F, member 15
−3.201877308	GLUL	glutamate-ammonia ligase
−3.201034593	PDGFRL	platelet-derived growth factor receptor-like
−3.178365791	PTHLH	parathyroid hormone-like hormone
−3.140789142	LRRC8D	leucine rich repeat containing 8 family, member D
−3.13580574	ETV1	ets variant 1
−3.126214513	ARHGAP26	Rho GTPase activating protein 26
−3.097254116	DSEL	dermatan sulfate epimerase-like
−3.081146219	KITLG	KIT ligand
−3.07957473	LOXL1	lysyl oxidase-like 1
−3.079257054	C1R	complement component 1, r subcomponent
−3.078946977	CLGN	calmegin
−3.076076876	EPHB2	EPH receptor B2
−3.033006625	PDE4DIP	phosphodiesterase 4D interacting protein
−3.028665038	SESN3	sestrin 3
−3.013137184	ABCA6	ATP binding cassette subfamily A member 6
−3.006587742	JAM2	junctional adhesion molecule 2
−3.000300304	EGFL6	EGF-like-domain, multiple 6
−2.950662537	GAS1	growth arrest-specific 1
−2.948913666	TWIST2	twist family bHLH transcription factor 2
−2.942970085	GPNMB	glycoprotein (transmembrane) nmb
−2.940267291	TGFBR3	transforming growth factor beta receptor III
−2.926118857	ASS1	argininosuccinate synthase 1
−2.923103244	FBLN5	fibulin 5
−2.908155453	CPED1	cadherin-like and PC-esterase domain containing 1
−2.905037264	SOD2	superoxide dismutase 2, mitochondrial
−2.880197455	STEAP1B	STEAP family member 1B
−2.875575087	GCNT1	glucosaminyl (N-acetyl) transferase 1, core 2
−2.848917057	CTTNBP2	cortactin binding protein 2
−2.837655832	LTBP4	latent transforming growth factor beta binding protein 4
−2.837440498	PRKCE	protein kinase C, epsilon
−2.83680985	C9orf64	chromosome 9 open reading frame 64
−2.824016788	SIX1	SIX homeobox 1
−2.791328188	TMEM119	transmembrane protein 119
−2.789168074	PAX9	paired box 9
−2.789134846	PCDHGC	protocadherin gamma subfamily
−2.786844273	FHOD3	formin homology 2 domain containing 3
−2.780629853	DPYD	dihydropyrimidine dehydrogenase
−2.7804381	LAMA2	laminin, alpha 2
−2.777579249	WNT2B	wingless-type MMTV integration site family, member 2B
−2.775503154	LAMA3	laminin, alpha 3
−2.748974947	PTPRQ	protein tyrosine phosphatase, receptor type, Q
−2.742645094	TBX3	T-box 3
−2.742105627	TRIM16L	tripartite motif containing 16-like
−2.739459433	EYA2	EYA transcriptional coactivator and phosphatase 2
−2.739101353	PPARG	peroxisome proliferator-activated receptor gamma
